# microRNAs Regulate Cellular Magnesium by Tuning Expression of the Plasma Membrane Protein CNNM4

**DOI:** 10.1021/acschembio.5c00296

**Published:** 2025-08-27

**Authors:** Tomas S. Lazarou, Helia Dehghan Harati, Lara K. Mahal, Daniela Buccella

**Affiliations:** † Department of Chemistry, 5894 New York University, New York, New York 10003, United States; ‡ Department of Chemistry, 3158 University of Alberta, Edmonton, Alberta T6G 2G2, Canada

## Abstract

Cyclin M4 (CNNM4) is a plasma membrane Mg^2+^ transporter that is important for the regulation of cellular and organismal Mg^2+^ homeostasis. CNNM4 is overexpressed in liver diseases, including non-alcoholic steatohepatitis and acetaminophen-induced liver injury, leading to aberrant levels of Mg^2+^. The regulatory mechanisms underlying the overexpression of this protein, however, remain unknown. Here, we demonstrate regulation of CNNM4 and cellular magnesium levels by microRNA. Using a high-throughput assay, we established the complete miRNA binding profile of the 3′UTR of the gene. Both upregulation and downregulation of CNNM4 were observed, though upregulatory activity appears more prominent. We demonstrate that in both cases, the effects arise from direct binding of the miRNAs to the CNNM4-3′UTR, and we provide direct evidence that they result in changes of cellular concentration of the metal in hepatocytes. The results reveal a potential role of microRNAs in the alteration of magnesium homeostasis in the liver through CNNM4 and, based on the miRNA proxy hypothesis, predict a broader role of CNNM4 and aberrant magnesium levels in breast cancer and other pathologies.

## Graphical abstract

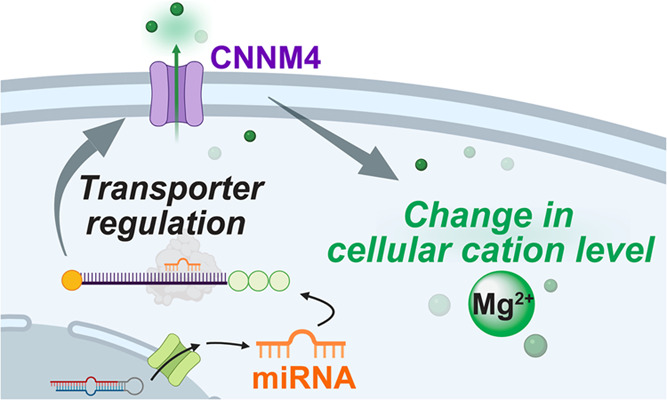

## Introduction

Magnesium­(II) is a metal cation required for numerous biochemical processes that are essential for normal cell function.^1,2^ Among its various roles, Mg^2+^ serves as a cofactor for over 600 enzymes, is involved in cellular signaling, and provides structural stabilization for a range of biomolecules.^1–3^ Because of its importance, the concentration of cellular Mg^2+^ is tightly controlled through the combined action of transporters that mediate the uptake of metal from the extracellular environment, export from the cytosol, and movement between organelles.^1^ Aberrant expression of magnesium transporters has been implicated in human disease, ranging from pancreatic,^4^ prostate,^5^ liver,^6,7^ gastric,^8^ and breast^9,10^ cancers to metabolic and liver diseases.^11,12^ The origin of such changes in transporter expression, however, remains unidentified in most cases.

Cyclin M4 (CNNM4) is a mammalian plasma membrane Mg^2+^ transporter that is responsible for maintaining Mg^2+^ homeostasis at both the cellular and organismal levels. It is predominantly expressed in the brain, intestine, and kidneys.^13,14^ Clinically, deleterious genetic mutations in *CNNM4* result in Jalili syndrome, characterized by cone-rod dystrophy and amelogenesis imperfecta.^15,16^ Upregulation of CNNM4 in liver cells has been recently linked to acetaminophen-induced liver injury (AILI)^12^ and to the development of Non-Alcoholic Steatohepatitis (NASH),^11^ which contributes to the incidence of hepatocellular carcinoma (HCC). Aside from recent efforts to investigate the interactions of CNNM proteins with various binding partners to regulate their activity,^10,17–23^ the regulation of CNNM4 expression itself remains to be investigated.

Research on the regulation of gene expression for metal transporters has largely focused on transcriptional regulation, with less attention given to post-transcriptional modes of regulation such as microRNAs (miRNAs).^24,25^ miRNAs are small noncoding RNAs that are ∼20–30 nucleotides long and modulate protein translation by binding to the 3′-untranslated region (3′-UTR) of mRNA.^26^ miRNA post-transcriptional regulation is thought to occur in one of two ways: (i) the miRNA promotes deadenylation of the mRNA transcript, which leads to mRNA degradation; or (ii) the miRNA halts the translation machinery as it is reading the transcript, thus repressing protein expression. More recently, miRNAs have also been found to enhance protein expression through direct binding to the 3′-UTR.^27–29^ The effect of miRNAs on gene expression levels is typically not extreme on/off; rather, miRNAs fine-tune the expression of proteins in the cell^30^ and are thus well poised to adjust the levels of essential metal transporters in a coordinated fashion as cells shift their homeostatic setpoints in disease. This possibility, however, has not been thoroughly explored.

Abnormal levels of Mg^2+^ have been linked to HCC,^31–33^ and miRNAs have been shown to play a direct role in the progression of this and other liver diseases.^34–38^ Yet, to date, there are few examples of miRNA regulation of genes implicated in magnesium homeostasis in humans, and the connection with liver disease has not been investigated. miR-135a has been shown to downregulate transient receptor potential melastatin 7 (TRPM7), a plasma membrane Ca^2+^ and Mg^2+^ transporter, in neonatal rat cardio fibroblasts.^39^ More recently, miR-199a-5p was shown to downregulate the expression of MagT1 in HBV-infected CD8^+^ T cells^40^ and, during the course of our studies, miR-24-2-5p was shown to regulate CNNM4 in models of metastatic breast cancer.^41^ These examples point to a potential role of miRNAs in regulating Mg^2+^ levels in the cell, though those studies only demonstrated changes in mRNA and protein levels and did not explore phenotypic changes in metal content. Herein, we present the first comprehensive experimental profiling of miRNAs targeting CNNM4 and demonstrate both post-transcriptional downregulation and upregulation of this transporter in hepatic cancer cells. Beyond protein levels, we demonstrate the effects of miRNA-mediated regulation on basal cellular levels of free Mg^2+^, thus revealing a possible mechanism for the reset of the homeostatic setpoint in liver disease.

## Results

### High-Throughput Analysis Identifies miRNA Regulating CNNM4 Expression

To examine miRNA regulation of CNNM4, we utilized the miRFluR assay, an *in cellulo* high-throughput miRNA-mRNA interaction screen previously developed by the Mahal lab.^27–29,42^ The miRFluR assay can probe the entire human miRome against a single gene of interest using a genetically encoded ratiometric fluorescent reporter plasmid (pFmiR, Figure S1A). For our assay, we cloned the most prevalent 3′-UTR transcript of CNNM4 (Gene ID: 26504, Figure S1B) downstream of Cerulean in the pFmiR plasmid (pFmiR-CNNM4). mCherry, encoded in the same plasmid, acts as an internal standard to provide a ratiometric output and control for nonspecific effects of miRNA and for transfection efficiency. In a typical run, the pFmiR-CNNM4 sensor and a library of miRNA mimics (Dharmacon miRIDIAN library v.12, 2756 miRNA) arrayed in triplicate in 384-well plates were cotransfected into HEK293T cells. After 48 h, the ratio of Cerulean to mCherry fluorescence (*F*_C_/*F*_R_) was measured (Figures 1A and S2). For each plate, the data were normalized to data from cells transfected with a nontargeting control (NTC) within the plate. After removal of miRNAs with a high error of the measurement (≥10%), we obtained 1572 miRNAs that were used to calculate *Z*-scores for the entire data set. miRNAs with *Z*-Scores ± 1.96 (two-tailed *Z* test, 95% confidence interval) were considered positive hits. A total of 70 miRNAs were found to significantly regulate the reporter gene, with 50 showing upregulation and 20 showing downregulation (Figure 1B,C. For a complete list of hits, see Table S2). Upregulatory miRNAs displayed a >1.4-fold increase in gene expression, while downregulatory miRNAs showed >2-fold repression in the selected confidence interval.

**Figure 1:**
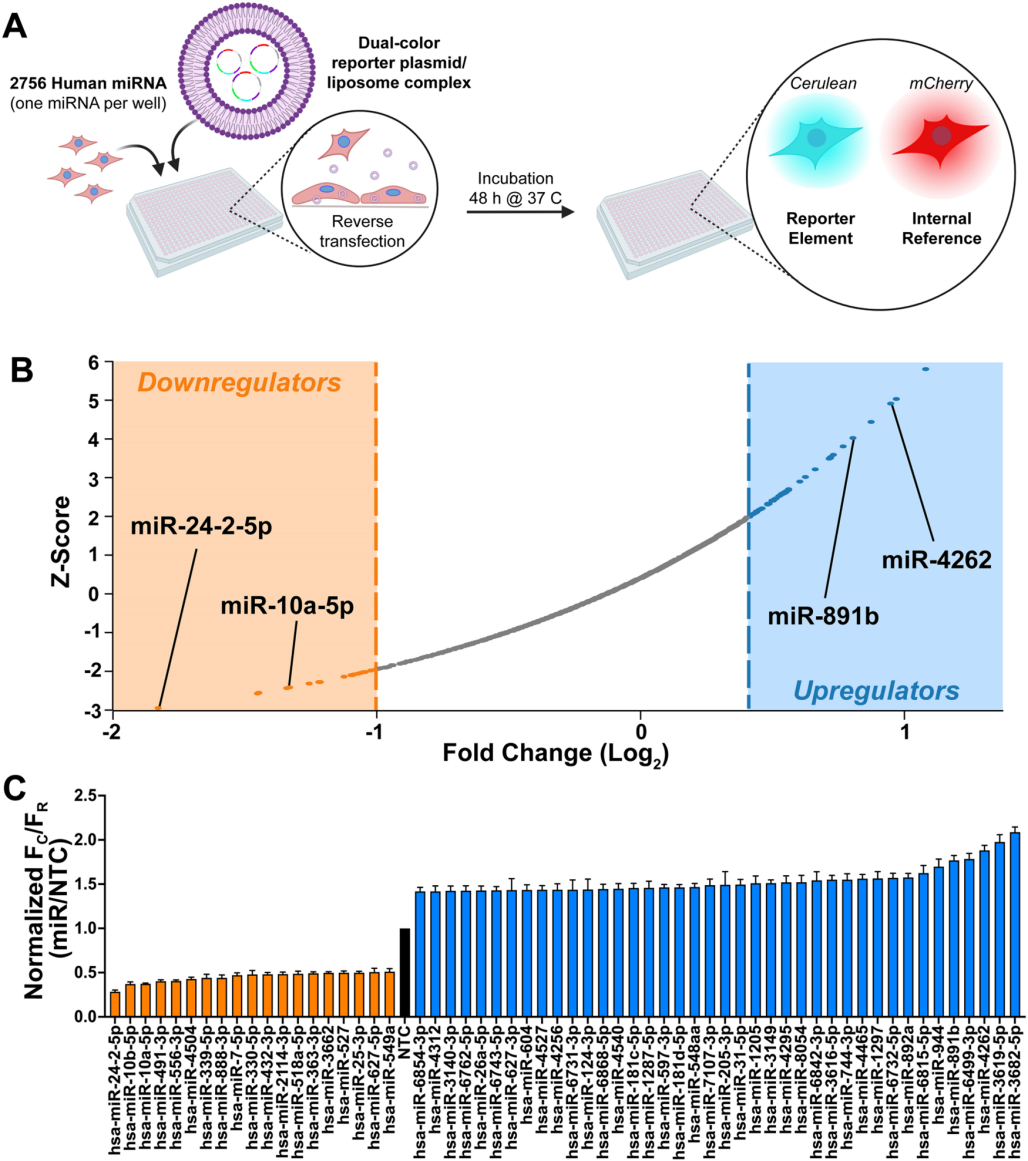
Identification of miRNA regulators of CNNM4. (A) Overview of miRFluR high-throughput screen of miRNA-mRNA interactions. Reverse transfection of both the pFMIR plasmid and the miRNA mimic allows for each miRNA to be interrogated for regulatory activity against CNNM4, based on a ratiometric fluorescence readout. (B) Results from miRFluR screen against the 3′UTR of CNNM4. Plot of Z-score vs Log_2_ (fold change, normalized against NTC) for miRNA passing quality control (1572 miRNA). miRNAs within the 95% confidence interval are highlighted (orange, downregulatory miRNAs; gray, controls/nonregulatory; blue, upregulatory miRNAs). (C) Complete list of hits identified in the screen. Error bars represent propagated error over three wells.

### miRNAs Regulate Endogenous CNNM4 Expression in Liver and Breast Cells

To validate the miRFluR results for CNNM4, a subset of miRNA hits was selected to test for the regulation of endogenous transporter expression. The chosen miRNA subset is comprised of two downregulatory miRNAs (miR-10a-5p and miR-24-2-5p) and two upregulatory miRNAs (miR-891b and miR-4262), all of which have shown differential expression in models of HCC compared to normal phenotypes.^43–49^ We posited that they might be relevant to the aberrant regulation of Mg^2+^ homeostasis observed in the disease.^31–33^ Transfection of hepatocellular carcinoma HepG2 cells with miRNA mimics of the selected hits resulted in changes in CNNM4 protein expression that parallel those obtained in the miRFluR assay (Figures 2A and S3). Both miR-10a-5p and miR-24-2-5p resulted in significantly lower expression of CNNM4, whereas miR-891b and miR-4262 resulted in a higher expression of the transporter. To assess the ability of miRNAs to affect CNNM4 expression more broadly across cell types, we also tested the selected miRNA mimics in the breast cancer cell line MCF7 (Figures 2C and S3), which shows robust endogenous expression of CNNM4.^50,51^ Both miR-10a-5p and miR-24-2-5p significantly reduced CNNM4 expression, while only miR-4262 significantly upregulated CNNM4 in MCF7 cells.

**Figure 2:**
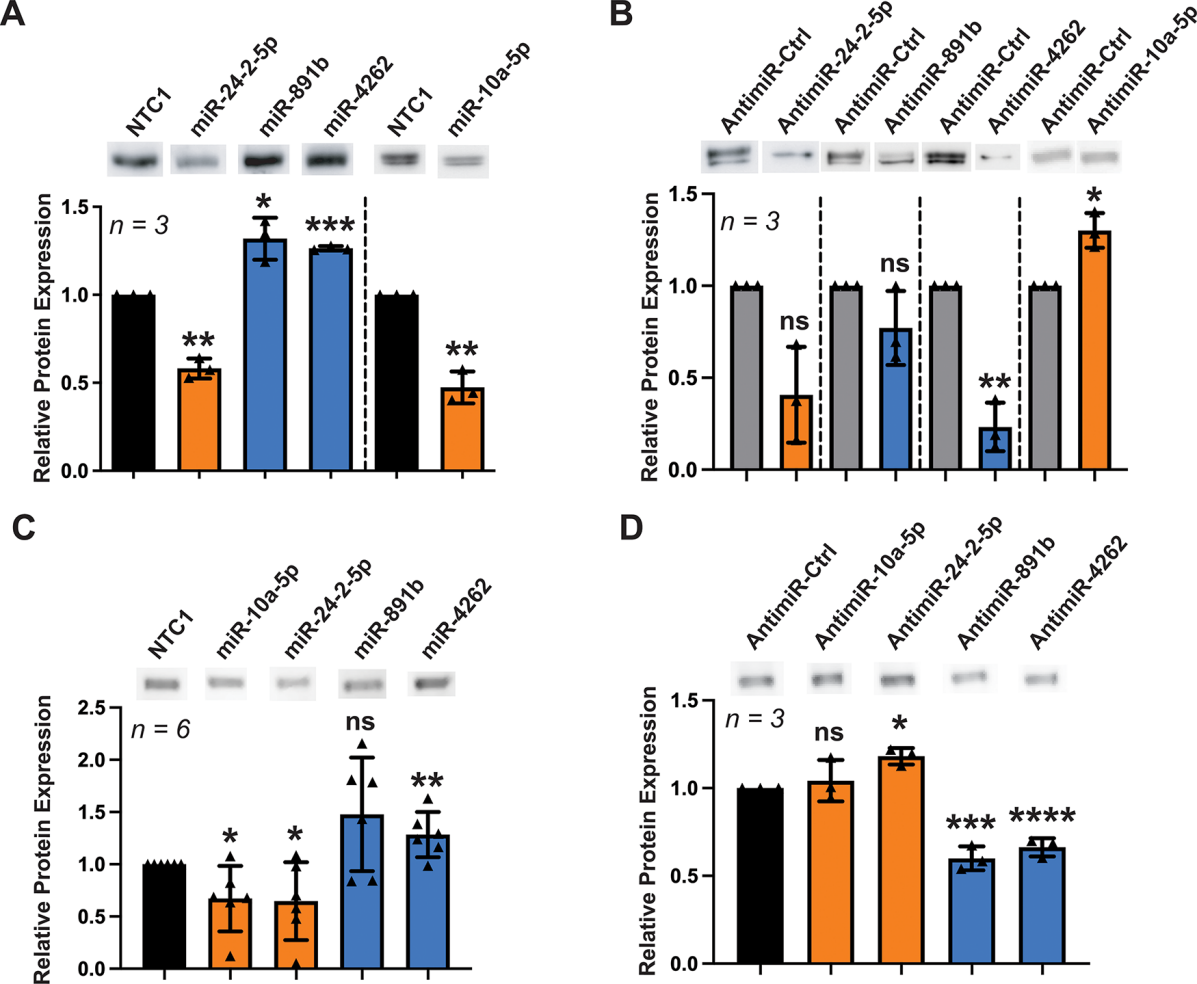
Validation of miRNA targeting of CNNM4. (A) Quantification of Western blots of CNNM4 from HepG2 cells transfected with miRNA mimic or nontargeting control (NTC). Representative blots are shown above each bar. Protein signal was normalized against total protein content (Ponceau S) and reported relative to the signal from the NTC. Downregulatory miRNA shown in orange; upregulatory miRNA shown in blue. The dotted line indicates separate experiments normalized to different NTC. (B) Quantification of Western blots of CNNM4 from HepG2 cells transfected with anti-miRs or an anti-miR control. Processed in the same manner as the miRNA mimics. (C) Quantification of Western blots of CNNM4 from MCF7 cells transfected with miRNA mimic or nontargeting control (NTC). Processed in the same manner as the HepG2 data. (D) Quantification of Western blots of CNNM4 from MCF7 cells transfected with anti-miRs or an anti-miR control. Processed in the same manner as the HepG2 data. (*P < 0.1; **P < 0.01; ***P < 0.001; ****P < 0.0001; ns, not significant).

To investigate the regulation of CNNM4 by endogenous miRNAs, HepG2 and MCF7 cells were then transfected with miRNA inhibitors (anti-miRs) corresponding to the selected miRNAs (Figure 2B,D). As expected, transfection of anti-miR-4262 resulted in a significant decrease in CNNM4 expression in both HepG2 and MCF7 cells from inhibition of the upregulatory miRNAs. Transfection of anti-miR-10a-5p in HepG2 cells resulted in the expected increase in CNNM4 expression. Transfection with anti-miR-24-2-5p and anti-miR-891b, on the other hand, resulted in no significant changes in the expression of the transporter in HepG2 cells. This may stem from low endogenous levels of the corresponding miRNAs in HepG2 cells,^44,49^ resulting in changes too small to detect. In MCF7 cells, both anti-miR-891b and anti-miR-24-2-5p produced their expected change, while anti-miR-10a-5p resulted in a small but nonstatistical increase in CNNM4 expression.

We next sought to determine whether the observed effects result from direct interaction of the miRNA with the mRNA of the target gene. To this end, we predicted computationally the binding site of selected miRNAs on the 3′-UTR of CNNM4, and then mutated the sites within pFmiR-CNNM4 to assess the effect experimentally. For upregulatory miR-891b and miR-4262, we used the miRNA binding prediction program RNAHybrid and selected the predicted sites with the highest binding energy^52^ (−22 and −20 kcal/mol, respectively, Figure 3A,B). Both sites exhibit noncontiguous binding and are not AU-rich, supporting claims that upregulatory miRNAs primarily bind through noncanonical interactions.^27–29^ For both miRs, mutation of the predicted site to the complementary sequence in the pFmiR sensor abolished upregulation of cerulean expression upon cotransfection of the concomitant miRNA mimics, providing strong evidence that regulation is mediated by direct interactions at these sites (Figure 3A,B). For downregulatory miR-10a-5p, we used TargetScan to predict the binding site to the 3′UTR of CNNM4 (Figure 3C). The potential site contained a 7 base pair seed region, one base away from the 5′ end of the miRNA, in line with canonical miRNA binding sites.^53–55^ Mutation of this site abrogated the impact of miR-10a-5p on the cerulean expression from the pFmiR-CNNM4 sensor (Figure 3C). Interestingly, mutation of this site also abrogated the impact of miR-10b-5p (Figure S4A), a family member of miR-10a-5p that was also identified as a downregulator in our screen. The two miRNAs are predicted to target the same site and share seed sequences. miR-10b-5p showed significant downregulation of CNNM4 expression in HepG2 cells (Figure S4B), though its anti-miR, like that for miR-10a-5p, resulted in a small but nonstatistical increase in expression (Figure S4C). In summary, the mutational studies indicate that observed changes in expression of CNNM4 result from the *direct* interaction of the miRNAs with the mRNA of the gene.

**Figure 3:**
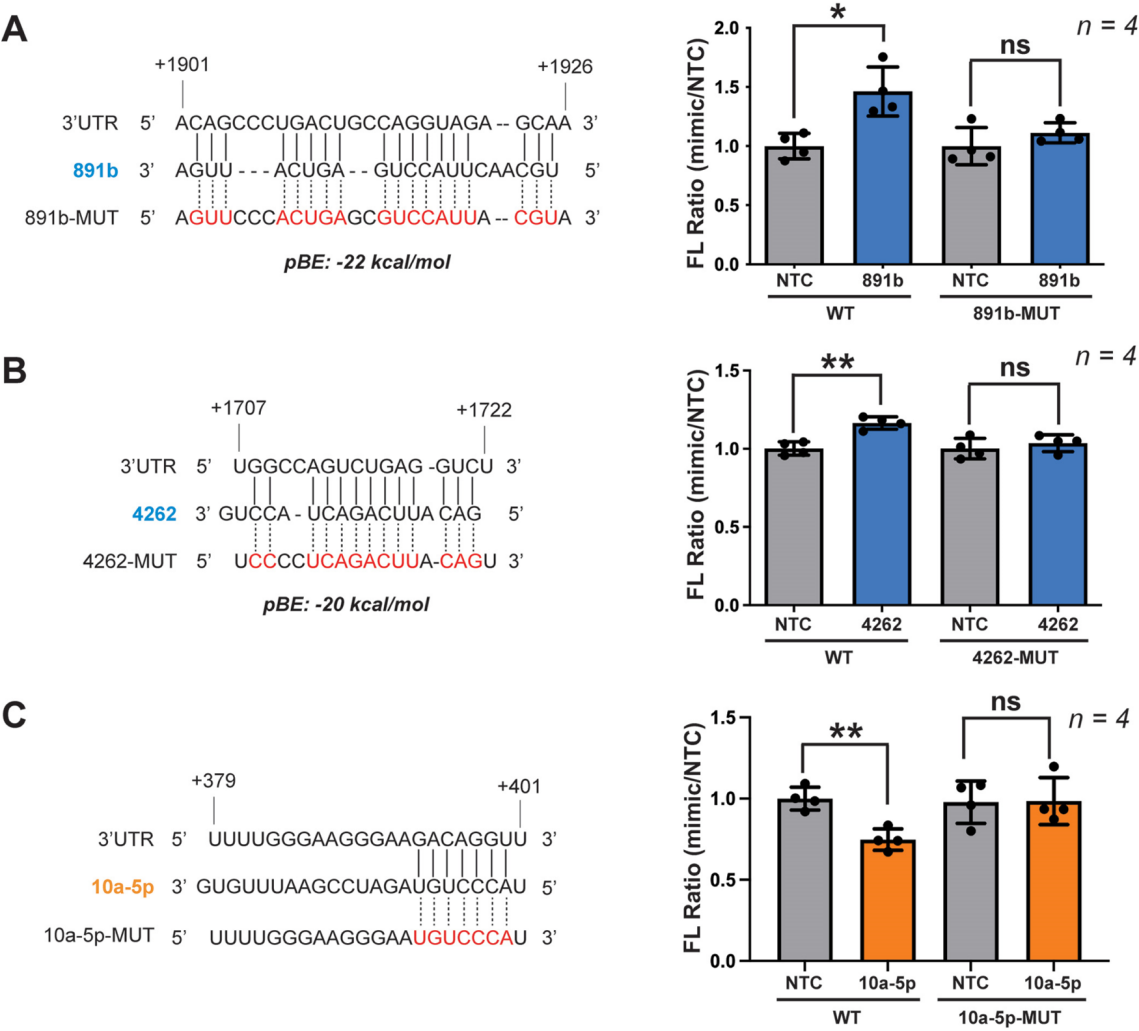
Predicted binding sites of selected miRNA in the 3′ UTR of WT CNNM4 and results of mutations introduced on the sites (mutated bases shown in red). Binding sites for upregulators miR-891b and miR-4262 (A, B) were predicted using RNAHybrid, and for downregulator miR-10a-5p (C) using TargetScan. Bar graphs show changes in expression of reporter genes in pFmiR-CNNM4 upon introduction of the shown mutations. Data were normalized over NTC for each sensor. Statistical analysis using the standard *t* test (*P < 0.1; **P < 0.01; ***P < 0.001; ns, not significant)

### miRNA Regulation of CNNM4 Results in Changes in Mg^2+^ Levels in Liver Cells

Work from our and the Martinez-Chantar laboratories have demonstrated that dysregulation of CNNM4 expression in various models of liver disease, including NASH and AILI, results in changes in cellular levels of free Mg^2+^.^11,12,56^ To interrogate the effect of miRNA modulation of CNNM4 on metal levels in HepG2 cells, we assessed intracellular levels of free Mg^2+^ by fluorescence microscopy using a ratiometric fluorescence sensor for Mg^2+^, MagS, developed previously in our group (Figure 4A).^57^ Based on evidence that CNNM4 mediates cellular Mg^2+^ export in liver cells,^11^ downregulation of this transporter by miRNAs is expected to result in an overall increase in intracellular Mg^2+^, whereas upregulation should result in a decrease (Figure 4A). In line with our expectations, transfection of a mimic of the upregulatory miR-4262 in HepG2 cells caused intracellular Mg^2+^ levels to significantly decrease compared to cells transfected with nontargeting control (Figures 4B and S5). Conversely, transfection with downregulatory miR-10a-5p resulted in an increase in cellular levels of free Mg^2+^ compared to NTC, also consistent with the observed changes in CNNM4 expression. Transfection of the upregulatory miR-891b, however, resulted in an increase in intracellular Mg^2+^ concentration, whereas treatment with the downregulatory miR-24-2-5p resulted in a decrease in intracellular concentration of the metal, opposite the expected changes based on CNNM4 upregulation alone. These results may arise from miR-891b and miR-24-2-5p targeting other genes that may impact overall cellular Mg^2+^ homeostasis in conjunction with the upregulation of CNNM4 shown at the protein level.

**Figure 4:**
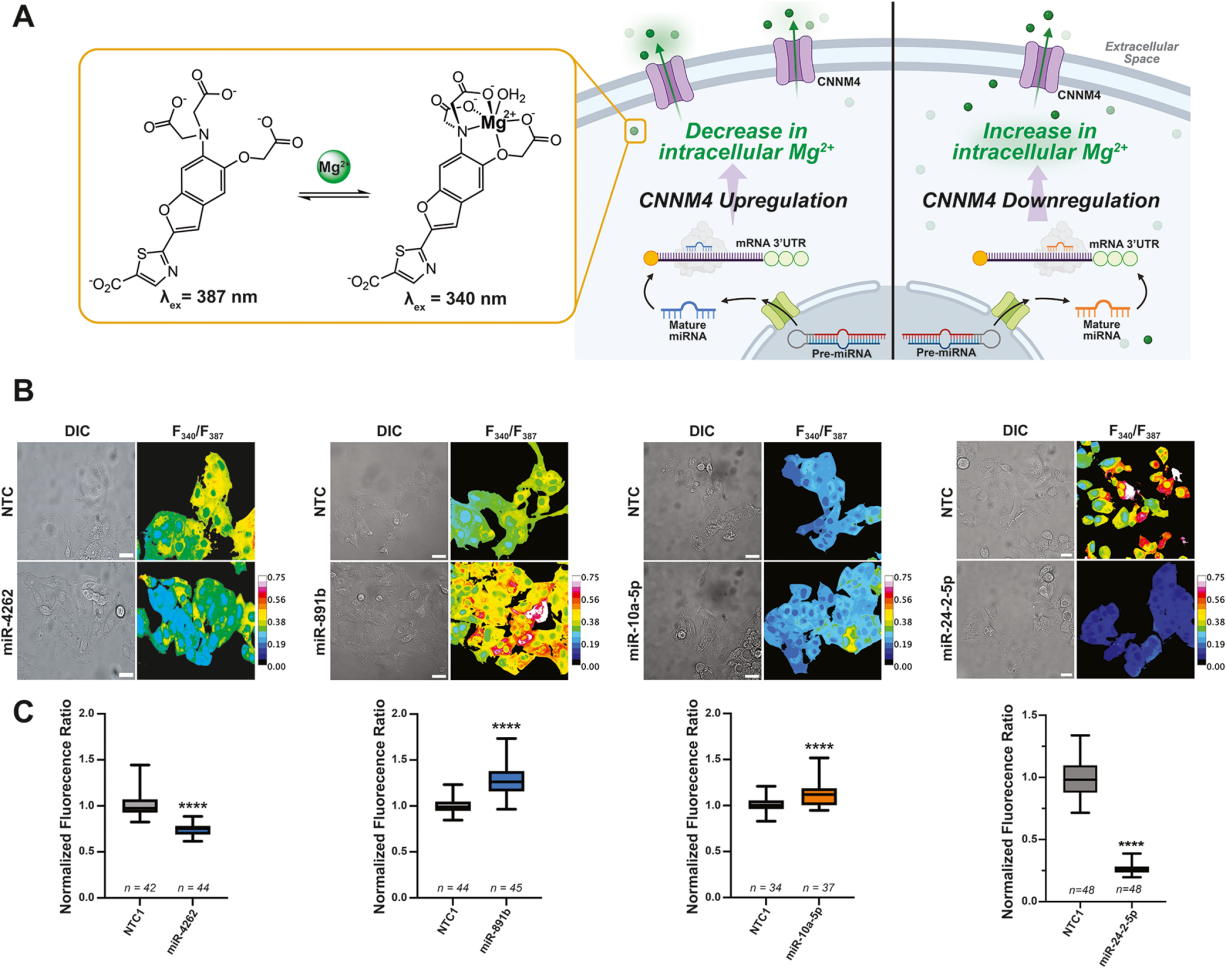
Changes in intracellular free Mg^2+^ in liver cells in response to miRNAs, revealed by fluorescence sensing of the metal. (A) Structure of the excitation ratiometric sensor for free Mg^2+^, MagS (applied to live cells in a membrane-permeable form, MagS-AM). Upon binding Mg^2+^, the excitation maximum of the sensor shifts and the fluorescence ratio, *F*_340_/*F*_387_, increases. Upregulation of CNNM4 results in a decrease in intracellular Mg^2+^; downregulation results in an increase of the metal. (B) Representative fluorescence microscopy images of live HepG2 cells transfected with miRNA-4262, miR-891b, miR-10a-5p, or miR-24-2-5p mimic and stained with MagS-AM. Respective controls transfected with a nontargeting miRNA shown. Scale bar = 20 μm. (C) Comparison of average fluorescence ratio per cell treated with selected miRNAs vs those treated with NTC under the same conditions. MagS ratios were normalized to the average NTC1 ratio in each trial. Statistical analysis using a standard *t* test with Welch’s correction. (****P < 0.0001).

## Discussion

The expression levels of transporters and channels have a critical effect on the membrane permeability to metal cations and overall metal homeostasis setpoints; abnormal expression patterns, in turn, can lead to aberrant cellular function and the onset of disease. CNNM4 expression levels in colon cancer have been demonstrated to be inversely correlated to colon cancer malignancy, suggesting that control of Mg^2+^ homeostasis by CNNM4 is important for colon cancer progression.^17^ Work from our and the Martinez-Chantar group has also demonstrated that CNNM4 upregulation leads to chronic depletion of intracellular Mg^2+^ in models of NASH and AILI,^11,12,56^ whereas silencing of the gene leads to restoration of cation levels and reversal of the disease phenotype in animal models of both conditions. These observations suggest that Mg^2+^ balance plays an important role in sustaining liver health. Regulation of Mg^2+^ transporter expression has remained vastly underexplored, leaving an important gap in knowledge of the factors that influence the homeostatic setpoint of metals and their shift in disease. There are limited examples focusing on post-transcriptional regulatory elements, such as miRNAs.^39,41^ Herein, we demonstrate the regulation of CNNM4 by miRNAs, focusing our validation studies on miRNAs known to be dysregulated in hepatic disease.

The study of miRNA regulation of gene expression often relies on prediction algorithms such as TargetScan^53^ or miRWalk.^58^ Whereas these resources are useful for potential target identification, only ∼0.01% of all predicted miRNA-target interactions have been validated experimentally.^59^ In studies comparing experimental data to predicted miRNA-target interactions, it is estimated that as few as 16% of all target predictions correctly identify functional interactions.^42,60,61^ Previous studies on miRNA regulation of proteins related to Mg^2+^ transport^39,40^ made use of these prediction algorithms and low- to mid-throughput assays, which limited the breadth and reach of the work as it pertains to Mg^2+^ homeostasis. Higher throughput methods are an invaluable tool to gain deeper insight into how cells regulate the expression of Mg^2+^ transporters and, as a result, Mg^2+^ accumulation and distribution patterns. The miRFluR assay,^27–29,42^ able to probe potential interactions with all known miRNAs, allows post-transcriptional regulation mechanisms to be probed experimentally and at high throughput. Herein, we provide a comprehensive miRNA binding profiling of the 3′-UTR of CNNM4, the first example of such characterization for a protein involved in Mg^2+^ homeostasis and, overall, of any protein involved in cellular metal transport.

The profile of miRNA binding to the 3′-UTR of CNNM4, as revealed by the experimental miRFluR screen, shows both down- and upregulatory effects of miRNAs, with the latter being roughly 2-fold more prevalent in the list of hits. Historically, downregulation of gene expression was thought to be the primary mode of action for miRNAs in proliferating cells,^26^ and upregulation had only been shown in nondividing cells and mitochondria.^62–64^ Recently, Jame-Chenarboo et al. demonstrated miRNA upregulation of glycosylation enzymes ST6GAL1 and ST6GAL2 in actively dividing cells, suggesting that miRNA upregulation may be more commonplace than initially thought.^27^ Follow-up studies have identified upregulatory sites for CD98hc, ST3GAL1, ST3GAL2 ^28^ and CMAS.^29^ These works have also shown that upregulation by miRNA, like downregulation, controls the expression of proteins in functional networks. The results obtained for CNNM4 support these findings and challenge the idea that miRNA-mediated upregulation is limited to nondividing cells. The detailed mechanism for upregulation remains to be fully elucidated; however, it requires Argonaute 2 (AGO2), which is critical for miRNA-mediated regulation generally, and does not require TNRC6A (aka GW182), which is crucial for miRNA-mediated downregulation.^27,62^

Dysregulation of miRNAs in liver tissue has been related to NAFLD progression,^36,37^ fibrosis,^35^ and liver cancer.^34,38^ Of the miRNAs identified in our screen, we selected cross-referenced miRNAs that were specifically dysregulated in HCC. With regards to the latter, miR-10a has been shown to be downregulated in HCC tissues as well as in multiple hepatoma cell lines such as HepG2, HepG3, SMML7721, and SK-Hep-1, compared to normal liver tissues and cells.^43,48^ This is consistent with a net upregulation of CNNM4 in liver disease. The selected upregulators, miR-891b and miR-4262, have also been linked to HCC; for example, miR-891b has been shown to increase metastasis of SK-Hep-1 cells.^44^ In cellular models and HCC tissue samples, miR-4262 is upregulated compared to normal hepatic tissue and cell lines.^46^ Again, higher expression of this upregulatory miRNA in HCC is consistent with a net upregulation of CNNM4 and a potential role in this liver pathology.

The transfection of the selected miRNA mimics into HepG2 cells showed downregulatory and upregulatory effects on the CNNM4 level *in cellulo* consistent with those observed in the high-throughput screen. These results, combined with the studies on the mutated 3′UTR, confirm that CNNM4 expression in cellular models of HCC is susceptible to fine-tuning by the *direct* effect of miRNAs. This regulatory effect is not unique to liver cells; transfection of miRNA mimics in MCF7 cells showed both down- and upregulatory effects at play. Significantly, our results are consistent with a very recent report that showed *CNNM4* being a target for downregulation by miR-24-2-5p in breast cancer cells, with implications in early stages of bone metastasis.^41^

Our fluorescence microscopy experiments using Mg^2+^ sensitive indicators revealed that intracellular Mg^2+^ levels change in response to the changes in CNNM4 expression exerted by the miRNA. These experiments offer, to the best of our knowledge, the first direct demonstration of modulation of cellular magnesium by miRNAs. For the upregulatory miRs, the increase in the CNNM4 level would lead to a decrease in cellular Mg^2+^, whereas the downregulators would cause an increase in intracellular Mg^2+^ levels. The downregulation of miR-10a-5p that characterizes HCC compared to normal cells^43,48^ would imply a net depletion of Mg^2+^ in liver cancer. Conversely, miR-4262 upregulation shown in models of HCC^46^ would also have a net effect of decreasing cellular Mg^2+^. Taken together, these data predict magnesium downregulation in HCC similar to that previously observed in NASH and AILI.

It is important to note that even though changes in CNNM4 expression and Mg^2+^ levels in response to the tested miRNA are apparent, miRNAs identified in our screen may target multiple proteins with similar or competing effects on cellular Mg^2+^ accumulation. For example, miR-24-2-5p showed a high downregulatory effect on CNNM4 in our screen (Figure 2A) yet resulted in a marked decrease in Mg^2+^ *in cellulo* as revealed by a ∼74% decrease in fluorescence ratio with our Mg^2+^ probe (Figure 4B). This effect could be a result of target promiscuity and illustrates the need for more thorough profiling of miRNA and their targets. In this regard, high-throughput miRNA-target identification screens have shown that miR-744-3p and miR-10a-5p, herein identified to have upregulatory and downregulatory effects on CNNM4, respectively, also target Mg^2+^ transporter TRPM7.^65,66^ Furthermore, some of the other regulatory miRNAs identified in our screen are predicted to target the 3′-UTR of other members of the CNNM family (TargetScan predictions; see Table S3). These results emphasize the need for a more comprehensive study of miRNA effects on other genes implicated in Mg^2+^ transport; broader characterization of these target networks may reveal further crossing points in the complex net of genes that control metal availability in physiological and pathological states.

Further analysis of the gene target networks using miRNet^67^ and validated miR-target interaction data from miRTarbase^59,68^ combined with phenotypic disease associations in DisGeNET^67,69^ (Figure 5 and Tables S4–S7), revealed that the downregulatory miRNAs identified in our screen target a multitude of genes associated with liver carcinoma phenotypes (Figure S6A). Similarly, the upregulatory miRNAs showed an enrichment of gene targets that are connected to both liver carcinoma and liver cirrhosis (Figure S6A). These results emphasize the role that miRNAs play in liver disease and support the notion of a broader role for CNNM4 in the development of liver pathologies.

**Figure 5:**
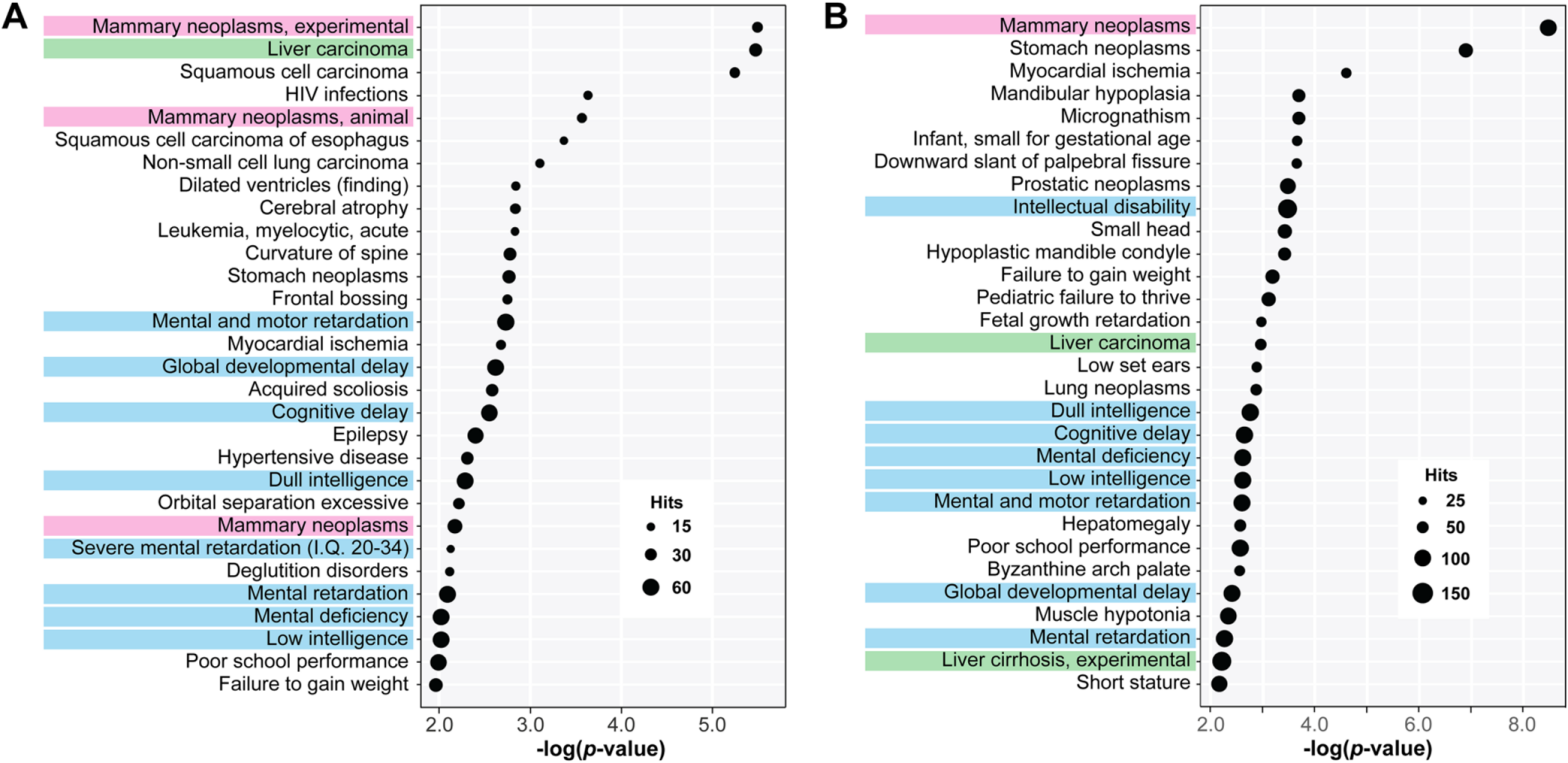
Phenotypic network analysis of miRNA regulators of CNNM4. Table of top 30 enriched disease phenotypes resulting from miRNet analysis of miRNA with downregulatory (A) and upregulatory (B) effects on CNNM4. Color coding of phenotypes of interest: liver cirrhosis/hepatocellular carcinoma, green; mammary neoplasms, pink; and genes related to impaired cognitive development, blue. Full list of enriched phenotypes available in Tables S6 and S7.

Significantly, the gene target network is enriched with genes linked to disease phenotypes beyond the liver. According to the miRNA proxy hypothesis, previously postulated by the group of Mahal and co-workers,^70–72^ these predict possible biological functions of CNNM4 and diseases in which its regulation, and that of Mg^2+^, may be relevant. For example, phenotypic analysis identified mammary neoplasms, cancerous and benign, as phenotypes associated with a large number of genes also targeted by the miRNAs that regulate CNNM4 (Figure S6B). While a clear connection between CNNM4 and breast cancer has yet to be established, *CNNM4* was recently identified as a gene regulated by miR-24-2-5p, a form of miR-24 proposed to confer a protective effect against breast-to-bone metastasis in early stages of breast cancer.^41^ *CNNM4* has been implicated in tumor progression across numerous cell types,^17,21^ and cellular Mg^2+^ levels have been shown to increase along with breast cancer proliferation.^2,73–75^ The decrease in cellular Mg^2+^ observed upon transfection with miR-24-2-5p mimics in our experiments points to a possible mechanism for the protective effect of this microRNA that warrants further exploration. To garner a more complete picture, the effect of this miR on other Mg^2+^ transporters, including protein binding partners and regulators, should be investigated.

The gene target network also points to other genes that may have a combined effect on Mg^2+^ cellular homeostasis, including phosphatases of regenerating liver PRL1 and PRL2 (PTP4A1 and PTP4A2, respectively). PRLs are known to associate with and inhibit CNNMs, countering their effect on Mg^2+^ cellular levels with particular relevance in oncogenesis.^10,17,76^ There are several reports of validated miRNA regulation of PRLs in the context of carcinogenesis;^77–84^ given the association of these phosphatases with CNNMs, it is plausible that these provide another pathway for miRNA regulation of Mg^2+^ cellular homeostasis in cancer. The limited studies to date have consistently reported protective effects of the miRNAs that downregulate these oncogenic phosphatases. Possible upregulation by miRNAs, however, has not been investigated yet and may reveal targets for therapeutic intervention.

Finally, phenotypes present in cognitive and developmental disabilities were also strongly associated with genes targeted by the same miRNAs identified in our screen (Figure S6C). CNNM4 has been reported to be highly expressed in brain tissue,^21^ but a link between CNNM4 and cognitive disorders has yet to be explored. Overall, the gene target analysis points to a broader role of CNNM4 and Mg^2+^ in human health and hints at connections that warrant further investigation.

## Methods

### General HEK293T Cell Culture and Imaging Protocols

HEK293T cells were cultured in Dulbecco’s Modified Eagle Medium (DMEM) supplemented with 10% fetal bovine serum (FBS) at 37 °C in a 5% CO_2_ humidified atmosphere. HEK293T cells were grown to 80% confluency in 10 cm culture dishes (Corning) and detached using 0.5% Trypsin without phenol red, neutralized with DMEM without phenol red, supplemented with 10% fetal bovine serum (FBS), 50 U/mL Penicillin, and 50 μg/mL Streptomycin. Cells were seeded at 12,500 cells/well in clear bottom, cell culture-treated black-walled 384-well plates (Corning) and after 24 h were transfected with 25 ng of pFmiR-CNNM4 construct using Lipofectamine 2000 (Life Technologies) at 0.1 uL per well. Cells were imaged 24 h after transfection. Fluorescence imaging experiments were performed on a Leica DMI6000B inverted fluorescence microscope equipped with a Hamamatsu ORCA-Flash 4.0 CCD camera, scanning stage, high-speed filter wheel for excitation filters, and a mercury metal halide external light source. For well imaging, a 10× objective was used along with a filter set for mCerulean and a Leica Texas Red filter cube for mCherry. The microscope was operated with Leica LAS AF software, and images were processed with ImageJ.^85^

### Cloning the pFmiR-CNNM4 Reporter Plasmid

An empty pFmiR plasmid^42^ (Figure S1A) served as the basis for cloning the 3′-UTR of CNNM4 into pFmiR. The 3′UTR sequence for CNNM4 was amplified from pLS luciferase vectors (SwitchGear Genomics), using primers (IDT) with the cut sites underlined (Table S1).

3′-UTR amplification was confirmed by agarose gel electrophoresis and purified by gel extraction (Promega) for further ligation procedures. The 3′-UTR of CNNM4 and empty pFmiR plasmid were digested with AgeI and BsiWI and ligated using the Q5 Hot Start High Fidelity DNA Polymerase (New England Biolabs). The sequences were confirmed by Sanger sequencing (Genewiz).

### miRFluR assay

The Human miRNA Mimic library version 21.0 + 19.0 (Dharmacon, Horizon Biosciences) was resuspended in nuclease-free water and aliquoted into black 384-well, clear optical bottom tissue-culture-treated plates (Nunc). Each plate contained three replicates of each miRNA (2 pmol/well).

Before each assay, the miRNA-containing plate was thawed at RT for 30 min and then centrifuged at 250 rcf for 2 min. The control miRNAs, NTC1 and miR-10a-5p (2 pmol/well), were added to empty wells on the plate in 5 μL of Opti-MEM (Gibco). Additional control wells containing HEK293T cells only had 15 μL of Opti-MEM (Gibco) added. To each well in the plate was added 25 ng of pFmiR-CNNM4 in 5 μL Opti-MEM (Gibco), and 0.1 μL lipofectamine 2000 (Invitrogen) in 5 μL Opti-MEM (Gibco). The solution was allowed to incubate at RT for 20 min. Then, HEK293T cells (25 μL per well, 500 cells/μL) in Dulbecco’s Modified Eagle Medium (DMEM, no phenol red) supplemented with 10% FBS, 50 U/mL Penicillin, and 50 μg/mL Streptomycin were added to the plate. Plates were then incubated at 37 °C under a 5% CO_2_ humidified atmosphere for 48 h. The fluorescence signals of Cerulean (λ_ex_: 433 nm; λ_em_: 475 nm) and mCherry (λ_ex_: 577 nm; λ_em_: 620 nm) were measured using the well-scan, bottom read option in a FlexStation 3 Multimode microplate reader (Molecular Devices).

### Data Processing for miRFluR Assay

*F*_C_/*F*_R_ for each well in each plate was calculated. For each miRNA, triplicate values were averaged, and the standard deviation (S.D.) was obtained. The *F*_C_/*F*_R_ value for each miRNA was then normalized to the *F*_C_/*F*_R_ value for NTC1 within that plate, and the error was calculated by error propagation. A percentage error for each miRNA was calculated, and any triplicate measurement with a percent error above 10% was excluded from further analysis. Any miRNA that had low signal/noise in any fluorescence channel, defined as any well measurement lower than the mean background (cells only) + 3 × SD of the background (cells only), were excluded. Data from all plates were then combined, and *Z*-scores were calculated. A *Z*-score of ±1.960, corresponding to a 95% confidence interval, was used as a threshold for significance.

### CNNM4-3′UTR-miRNA Binding Site Determination

Binding sites for the selected upregulators, miR-4262 and miR-891b, on the 3′UTR of CNNM4 were predicted using RNAHybrid.^52^ This prediction relies on the minimum free energy (MFE) of miRNA and the target mRNA sequence. The binding site for the downregulator, miR-10a-5p, on the 3′UTR of CNNM4 was predicted using Targetscan,^53^ which assesses binding based on seed binding. Predicted binding sites were mutated in the 3′UTR of CNNM4 using primers designed using NEBaseChanger ver. 1.3.3 (Table S1).

Site-directed mutagenesis was performed using the Q5 Site-Directed Mutagenesis Kit (NEB), following manufacturer instructions. The resultant mutant plasmids were then transformed into 5α competent *E. coli* cells (NEB) for amplification. Mutants were confirmed with Sanger sequencing. Subsequently, miRFluR assays were conducted simultaneously for both WT CNNM4-3′UTR plasmids and the mutated CNNM4-3′UTR sensors, as mentioned previously. Each miRNA mimic for miR-4262, miR-891b, and miR-10a-5p was cotransfected with plasmid sensor into HEK293T cells in a minimum of three wells alongside the NTC for comparative analysis. Two days after cotransfection, the mean *F*_C_/*F*_R_ ratio was calculated for NTC, miR-4262, miR-891b, and miR-10a-5p for each mutated and WT sensor. The data corresponding to each miRNA mimic was normalized to NTC, and mutated sensor data was compared to the WT sensor data.

### General HepG2 and MCF7 Cell Culture and Imaging Protocols

HepG2 cells were cultured in Eagle’s Modified Eagle Medium (EMEM) supplemented with 10% FBS at 37 °C in a 5% CO_2_ humidified atmosphere. MCF7 cells were cultured in Dulbecco’s Modified Eagle Medium Nutrient Mixture F12 (Ham), DMEM/F12(1:1) supplemented with 10% FBS at 37 °C in a 5% CO_2_ humidified atmosphere. Fluorescence imaging experiments were performed on a Leica DMI6000B inverted fluorescence microscope equipped with a Hamamatsu ORCA-Flash 4.0 CCD camera, scanning stage, high-speed filter wheel for excitation filters and a mercury metal halide external light source. For imaging of cellular magnesium, a 63× objective was used, along with a fura-2 filter set (Leica). The microscope was operated with the Leica LAS AF software. Image processing for ratio determination was performed with ImageJ software.^85^ For fluorescence ratio images, background correction and thresholds were applied to individual images prior to the ratio calculation.

### miRNA Mimic/Inhibitor Transfection and Western Blot

HepG2 cells were seeded in six-well plates (80,000 cells per well), cultured for 24 h, and transfected with miRNA mimics or miRNA inhibitors (50 nM, Dharmacon) using Dharmafect 1 (5 μL, Dharmacon). MCF7 cells were seeded in six-well plates (40,000 cells per well), cultured for 24 h, and also transfected with miRNA mimics and miRNA inhibitors (50 nM, Dharmacon) using Lipofectamine 2000 (5 μL, Thermofisher). Media was changed 24 h post transfection with EMEM supplemented with 10% FBS for HEPG2 and DMEM/F12(1:1) supplemented with 10%FBS for MCF7, and harvested 24 h later. HepG2 cells were then lysed in ice-cold RIPA buffer supplemented with protease inhibitors, and 50 μg of protein was run on SDS-PAGE. MCF7 Cells were then lysed in ice-cold RIPA buffer supplemented with protease inhibitors, and 10 μg of protein were run on SDS-PAGE. Standard Western Blot analysis using rabbit α-CNNM4 (Abcam, 1:1000) and goat α-rabbit-HRP (2°, 1:1000, Cell Signaling Technologies) was performed (antibody validation, Figure S7). Blots were developed using Clarity Western ECL substrate (Bio-Rad), and densitometry measurements were performed using ImageJ.^85^ Efficient transfection of miRNA mimics using the above protocols was confirmed with transfection of a fluorescently labeled miRNA mimic (Figure S8).

### General Protocol for MagS Indicator Loading and Imaging

HepG2 cells grown in complete growth media were plated in 35 mm glass bottom dishes (MatTek) and incubated with 1 μM Mag-S-AM and 0.02% Pluronic F-127 (Sigma) in FBS-free EMEM for 20 min at 37 °C in a 5% CO_2_ humidified atmosphere. The cells were washed two times with FBS-free EMEM, bathed in 1 mL of FBS-free EMEM and incubated for another 20 min at 37 °C to ensure complete de-esterification of the indicator. After de-esterification and prior to imaging, the cells were washed with 2 mL of HBSS with no divalent cations, and 1 mL of HBSS with no divalent cations was added for imaging procedures.

### Network Analysis Using miRNet

Downregulatory and upregulatory miRNAs (20 and 50 miRNAs, respectively, Figure 1C) were used as input in miRNet (mirnet.ca)^67^ using the following parameters: Organism, human miRs; ID type, miRbase ID; Targets, Genes (miRTarbase v 8.0). The Diseases Phenotype Enrichment function was used for Figure 5.

## Supplementary Material















## Notes

Supporting figures; complete list of miRNA hits from MiRFluR assay; lists of validated gene targets for miRNA hits, from miRTarbase; and lists of enriched disease phenotypes associated with CNNM4 down- and upregulatory miRNAs, from DisGeNET. (PDF)
Table S2. (XLSX)
Table S3. (XLSX)
Table S4. (XLSX)
Table S5. (XLSX)
Table S6. (XLS)
Table S7. (XLS)

## §

Department of Chemistry, Emory University, Atlanta, Georgia, United States
