# Supplementary material for: microRNAs Regulate Cellular Magnesium by Tuning Expression of the Plasma Membrane Protein CNNM4
Source: ACS Chem Biol. 2025 Aug 27;20(9):2159–70. doi: 10.1021/acschembio.5c00296 (PMC12455565; doi:10.1021/acschembio.5c00296)
Supplement: Supplementary file 1 [file cb5c00296_si_001.pdf]

**Supporting Information for:**

# microRNAs regulate cellular magnesium by tuning expression of the plasma membrane protein CNNM4

Tomas S. Lazarou[1], Helia Dehghan Harati[2], Lara K. Mahal[2,*], and Daniela Buccella[1,3,*]
[1] Department of Chemistry, New York University, New York, New York, USA
[2] Department of Chemistry, University of Alberta, Edmonton, Canada
[3] Current address: Department of Chemistry, Emory University, Atlanta, Georgia, USA

* To whom correspondence should be addressed: daniela.buccella@emory.edu or lkmahal@ualberta.ca

**Table of Contents**

**Table S1:** Sequences of oligonucleotides used in this study 3

**Figure S1:** Plasmid sensor generated and used for miRFluR assay 4

**Figure S2:** Representative images of HEK293T cells in miRFluR assay 5

**Figure S3:** Ponceau and Whole Western blots for data shown in Fig. 2 6

**Figure S4:** MiRNA mimic and AntimiR analysis of miR-10b-5p with predicted TargetScan binding site 7

**Figure S5:** Changes in intracellular free $Mg^{2+}$ in liver cells in response to miRNAs, revealed by fluorescence microscopy 8

**Figure S6:** miRNet gene networks showing miRNA identified as regulators of CNNM4 against genes from miRTarBase enriched for disease phenotypes from DisGeNET 10

**Figure S7:** Validation of antibody for CNNM4 11

**Figure S8:** Confirmation of miRNA transfection in HepG2 cells 12

**Table S1**: Sequences of oligonucleotides used in these studies.

| Oligonucleotide | Sequence (5’ → 3”) | Experiment | Supplier |
|---|---|---|---|
| pFmiR-CNNM4_AgeI (fwd) | CTATCCACCGGTGTGGACGAGACCACAACTC | Cloning | IDT |
| pFmiR-CNNM4_BsiWi (rev) | CACAATCGTACGGCCACAGCCACTTTTATCTC | Cloning | IDT |
| M891b-Forward | cgtccattacgtaAAGACATTGGTGGGGGTA | Cloning | IDT |
| M891b-Reverse | ctcagtgggaactCAGGAATATAGCTCTGCC | Cloning | IDT |
| M4262-Forward | acttacagtCCTAGCCATAGAACTGACTC | Cloning | IDT |
| M-4262-Reverse | ctgaggggaGGGGTAGGCATACTTGGT | Cloning | IDT |
| M10a-5p-Forward | ccatAAGGAACTTTATTTAAAAAAAAAATATTTTTTTC | Cloning | IDT |
| M10a-5p-Reverse | gacaCTTCCCTTCCCAAAATGA | Cloning | IDT |
| M10b-5p-Forward | ccatAAGGAACTTTATTTAAAAAAAAAATATTTTTTTC | Cloning | IDT |
| M10b-5p-Reverse | gacaCTTCCCTTCCCAAAATGA | Cloning | IDT |

Underlined sequences represent cut sites.

**A**

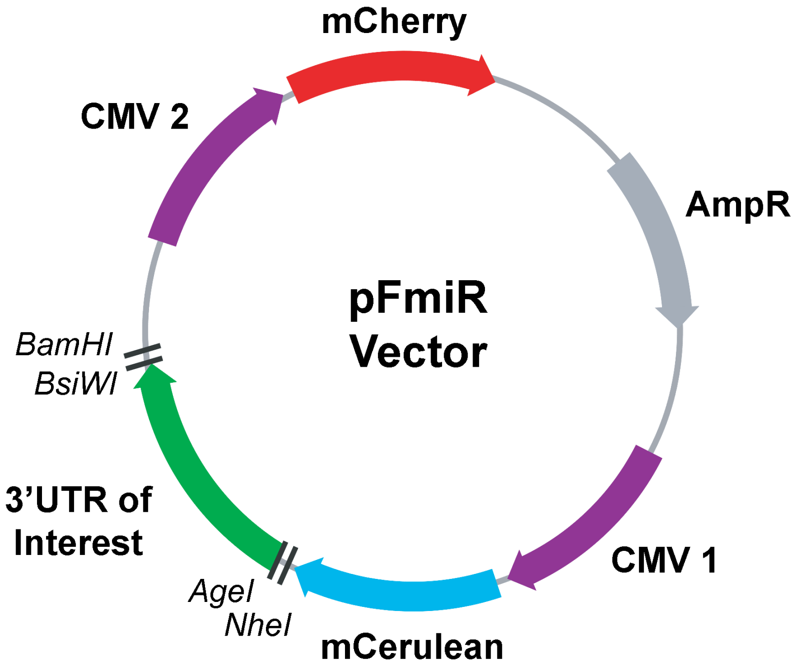


**B**

```
GTGGACGAGACCACAACTCTTCTCAACGAGCGTAACTCCTTGCTGCACAAAGCCTCCCACGAGA
ATGCCATCTGACAGGAGGGCCCGGGGCCCCCTGCCCACCCTGCGGGGGCCTCCCCAGTGGGCCC
ACATGAAGAGAGGGAACCTGTTAGTCCAGAAAGGATACGGATAGATAGCCTGTCTGACTGAACA
GCCAGATGGCCCCCAGCCTATGGGGGATCTGGCCTCTGCCAGGGACCTCTGAGTAGCTCTGAGG
TGGCACTGTCCAGCCCTGGATAGGGGGGGCAGTGGGCCAGCTACCGTAAGCAAAGGCTGTTTTT
TACTGAGAGAATTTCTAAAGTAGGCTCATCACTTTTTTTTAAATATCATTTTGGGAAGGGAAGA
CAGGGTTAAGGAACTTTATTTAAAAAAAAAATATTTTTTTCCTAAAAACTATAAAAGAGGAAGG
GTTTCTTGTCCCGGGAAGCAACGGACATAATCTGTTCCCAGCCATGGCCTTCCAGCTTGTGTCC
CTGATTCAGGGAGCTCTCCCTTCCTCCTCCTCCTCCTCCGGAGGTGGGATCCCAGAGCCTG
CCAGTGGAGGCTTATCTGTTGGGAGGAAGACAGCTCTTCACAGAAGCAAAGAACAAAATGGCAT
GGAGATCAGCTGCCTGAGCACCTGCGCTGTAGCTTATCTGACAACGCTGAGGCCACGAGCTCCT
GGGTAGCTGTGATCAGGGACATGATAATCTGAGCTATGCAGAGGAGCACATCTGTTGTCAACTG
CTGTACCCAGAAATCTAGAACTCTGCCGACAGCCTCTCCTGGTGAGTCGGGACTCAGCTGAGGA
CACATCCCCACCCTGCCTCCCATCTGGCCCTTTGGACAACTGGCCCTTTGTGACAGGGCTGACT
CAAGTGTTAGGCAGGGTCTCAGGCCTTTGATTGCTCACCCCTGCTCCCCAGGCCCTGCCCTCAC
TTTTACCAAAGGTTCTCCCTCGGCGGGAGGGCATCTGTGTTGGAGGTGATTTGTCTGGGTTCTT
CCTTTTGGTTCCAGAAGGAACTGTCAGTCATCAGCATCTGCGTTGTTAGCAGTCAGTACCACCC
CCGCCCCACAATGACAGTCAAGGCTGACTTGTTGACTGAAGCCTTTTTCCCAGACCCCTTATTT
CGAATCCCCAAGCTTCAGTCCCTCTTGGGGGTGGAGACAAGAGGACATGTGGGAAGCCACGGAA
GCAGGTTCTTTATGTCCTCTCCTCTGTGGCTGGCAAGGCTCACCTGGCCTTATCCACCCACTTA
TGGAACCTCAGGAGAGGAGGGCTCCTCCTAAAGGCATGCAGCTTGCAGCCCCTCTTTCTCACAC
GTGTGATCCTAGCGTGAGAGGTCATCCTGCCCTTGCTGAAGTTAGTAGTACTGTACTAAGAGCT
CTGCCCTCATGTGAATTCCTGCCCTGGCGCCTCTTCCCTGGGGCTGAATCAGGCCCTGCTGCAA
AACTCCAGGCTTCCCAGGGTTGGGGAGGCTGTGGGACCAAGGTCCATGTTGGTCCTTCCACTGG
GTGCAGCAGGAGCTGGGTCCCGAGAGCCTGGCAGGTGAAACTCTGCAGGCCTTCCGCCTGATTA
TTATTTATTCACTCCTTTCCTCACCCCAAGTGCCCTGCTCTCCAGGTGCCTAGAGTATCCTAAC
TCTTAGGACCAGGGATTGTCTTGCACCAAGTATGCCTACCCCTGGCCAGTCTGAGGTCTCCTAG
CCATAGAACTGACTCCTGGAAGCCTGGAGAGAAGGTGGTGACACCCATGGGTTCTCAACTGTAA
GGAAAAAAGACACCAGACTTTTGTTCCCTAGTGGGGGAAAGCCCTTAGTCTTGTACAGGAGCAG
CTTGCTCCCAAGTCCTTTTGGAAGCTGGCAGAGCTATATTCCTGACAGCCCTGACTGCCAGGTA
GAGCAAAAGACATTGGTGGGGGTATGTGAAGCAAAAGGGGCAGGTGCACACACCTCCACAGTGA
CCTCTGTGCACACGGTTACCACCAACTGGCTGGCCCTCCTCCTCTTCCCTGGCCCATTGATCAT
CCCTTCTCACAGAGGGTCATCATTATTTCCAAATATTGTTTGTCTGATGACTTCCTCTTCCCAG
TGCAATTTTTCCCTTCCTATTTCAACCTCTGGTTCCTGGGATGAGCCATACCCTGGAACTGGCC
CACCCACTGTGTCTTCCACGTAAGGGAGACCTTTGCAAAGGGCATCCAAATGGGTAGGCAGGTG
ACAGCCGCCGTATTTATTTTGCATAATATTTTAATTTGTATATTTTTGTGATTTATTTTGGCGT
TATGAGTTTGACTCTCGGGGAGTTTTGTTGTTATGACTCTTGTGTCTTTTGTCACAAAACAATG
ATATTTGCTAAACGATATATGGAATTTATTTTTGATTGGTAATAAAAAATCAAATATGTATAAA
TCCTGGTGAATCTACAACTTGCCTGTTTGTTCTGTCAGTATTCAGTATGTTGTTGAGATAAAAG
TGGCTGTGGC
```

**Figure S1. Plasmid sensor generated and used for miRFluR assay.** A) pFmiR-CNNM4 plasmid. B) 3'UTR sequence of CNNM4. Gene ID: 26504.

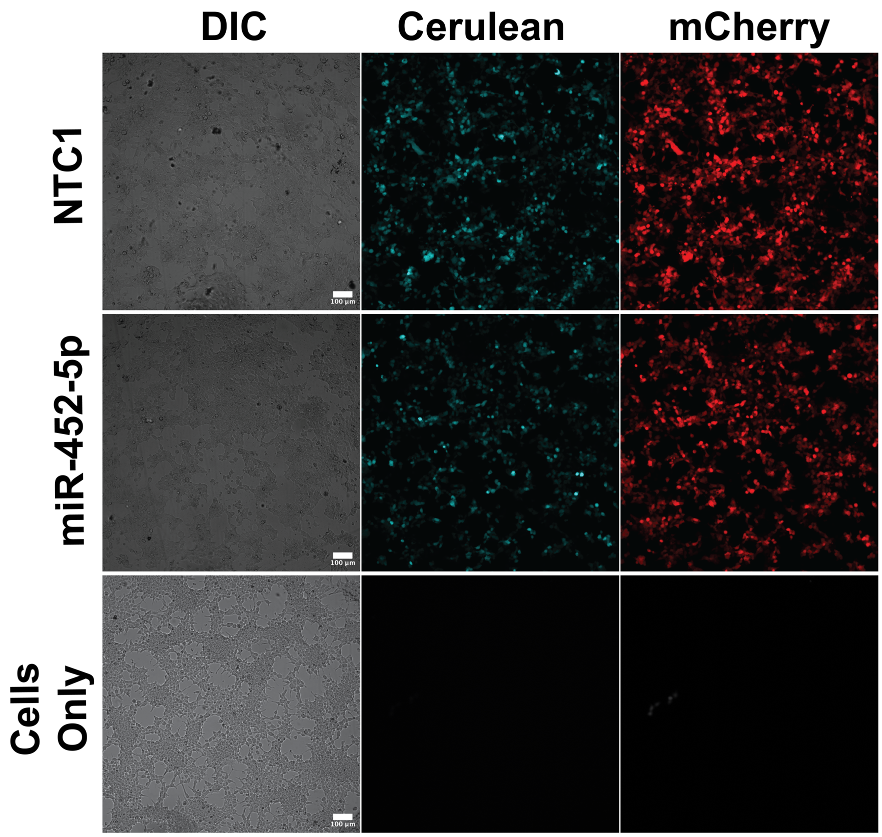


**Figure S2: Representative images of HEK293T cells in miRFluR assay.** Images of representative wells, collected 48 h after transfection with pFmiR-CNNM4 sensor and a miRNA mimic. Scale bar: 100 μm

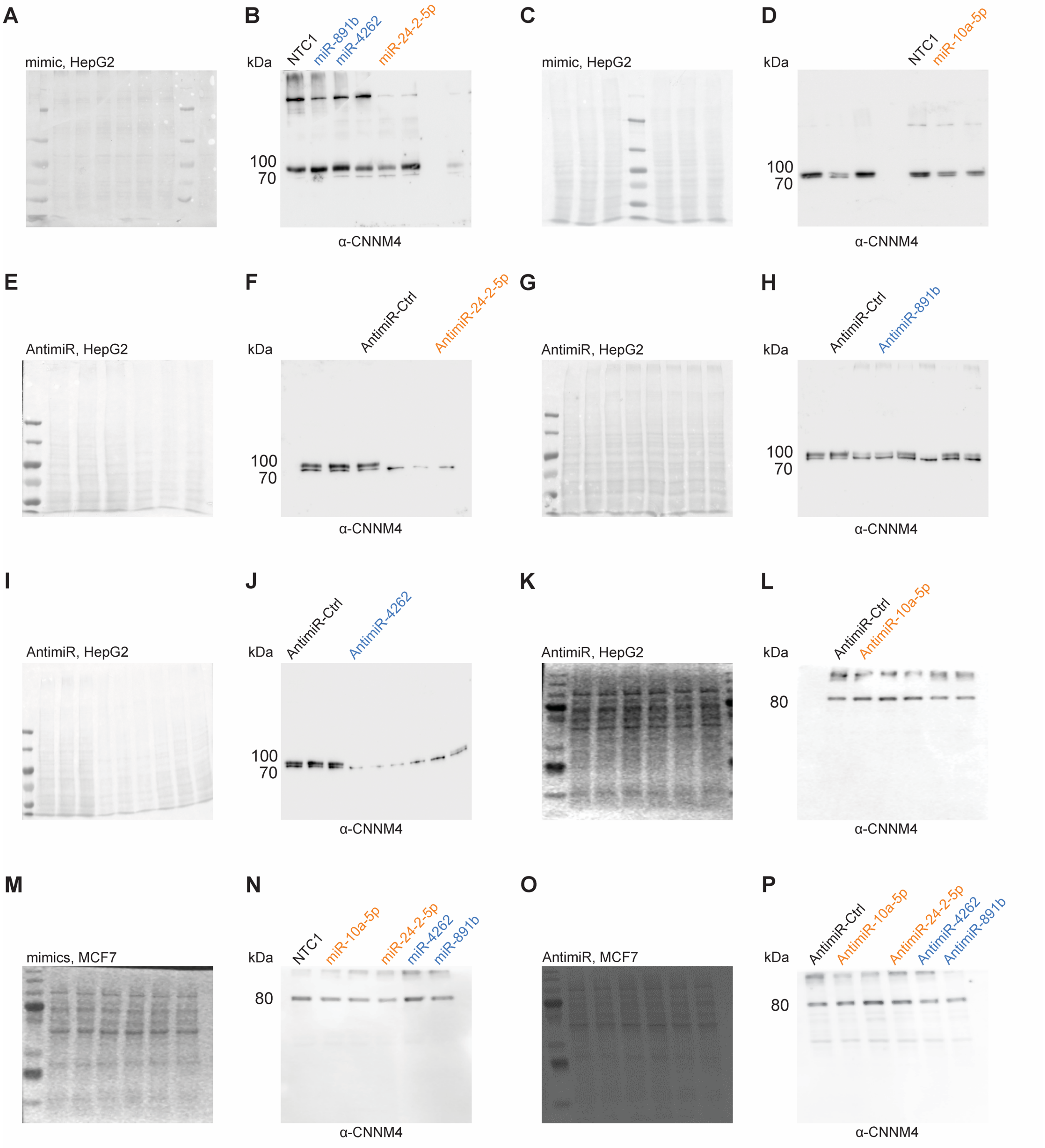


**Figure S3: Ponceau and Whole Western blots for data shown in Fig 2.** (A, C, E, G, I, K) Whole Western blot for data shown for HepG2 in Fig. 2A, B. (B, D, F, H, J, L) Ponceau staining of HepG2 blots used in Fig. 2A, B. (M, O) Whole Western blot for data shown for MCF7 in Fig. 2C, D. (N, P) Ponceau staining of MCF7 blots used in Fig. 2C, D.

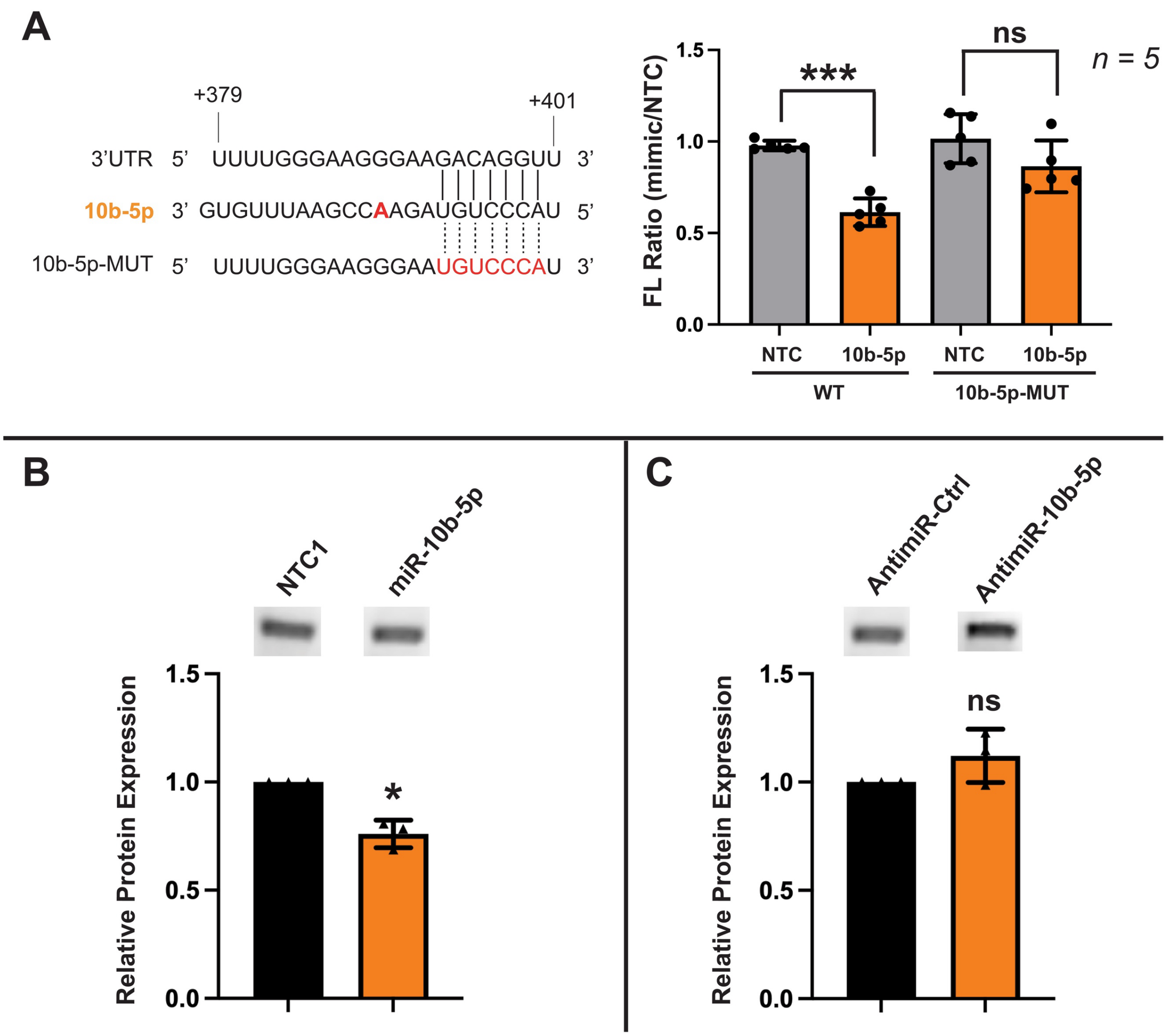


**Figure S4: MiRNA mimic and AntimiR analysis of miR-10b-5p with predicted TargetScan binding site. Related to Figure 2.** A) Quantification of Western blot of CNNM4 from HepG2 cells transfected with miR-10b-5p. Representative blot is shown above each bar. Protein signal was normalized against total protein content (Ponceau S) and reported relative to the signal from thwe NTC. B) Red base in miR-10b-5p sequence is the only differing residue compared to miR-10a-5p. Alignment of CNNM4 3'UTR with miR-10b-5p and its corresponding mutant. Mutated bases are shown in red. Bar graph data for mutant miR-FluR sensors. Data were normalized over NTC1 in each sensor. Statistical analysis using the standard t test compared the impact between wild-type and mutant sensors. C) Quantification of Western blots of CNNM4 from HepG2 cells transfected with anti-miRs or an anti-miR control. Processed in the same manner as the miRNA mimics. (*, $P < 0.1$; ***, $P < 0.001$; ns, not significant)

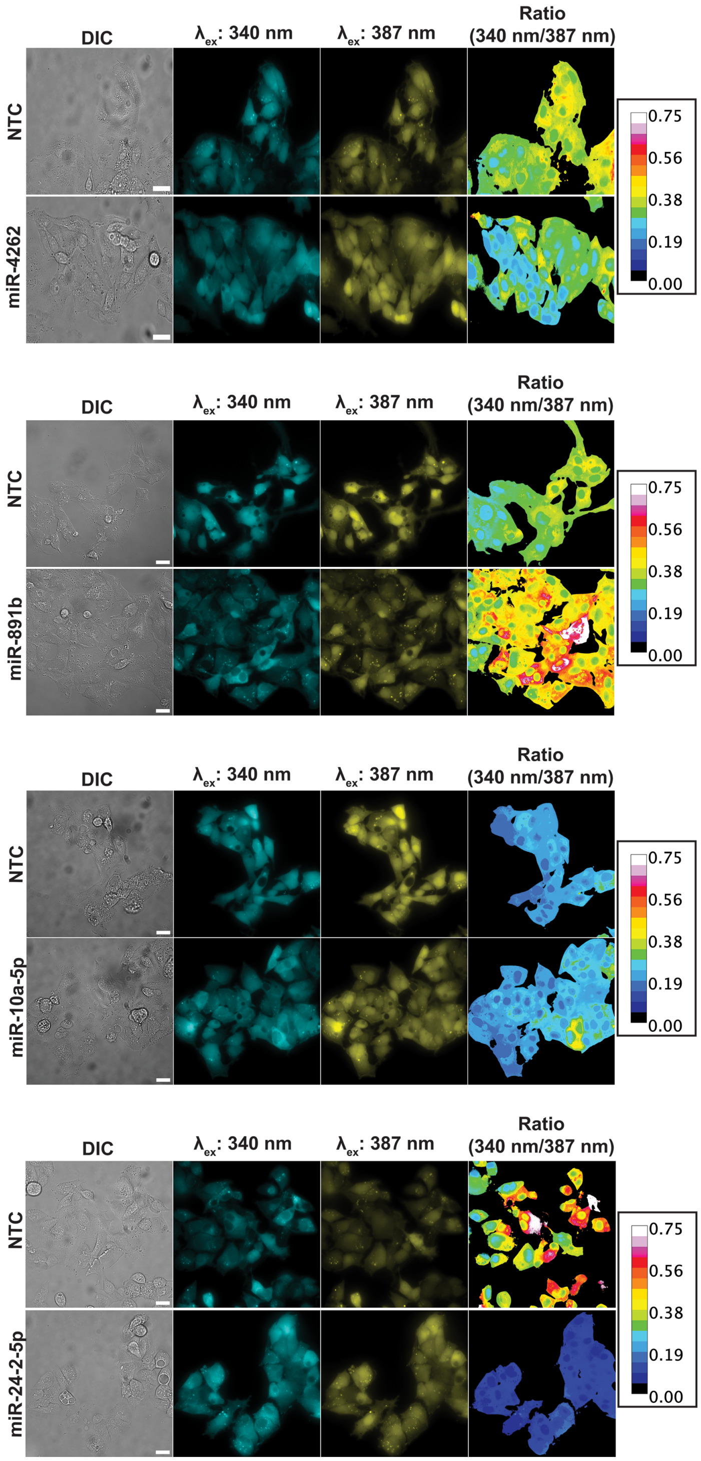


**Figure S5. Changes in intracellular free $Mg^{2+}$ in liver cells in response to miRNAs, revealed by fluorescence microscopy. Related to Figure 4.** Representative fluorescence microscopy images of live HepG2 cells transfected with miRNA-4262, miR-891b, or miR-10a-5p mimic and stained with MagS-AM. Respective controls transfected with a non-targeting miRNA shown. $\lambda_{ex}$: 340 nm represents the $Mg^{2+}$ -bound species of MagS, $\lambda_{ex}$: 387 nm represents the $Mg^{2+}$-unbound species of MagS. Scale bar = 20 µm.

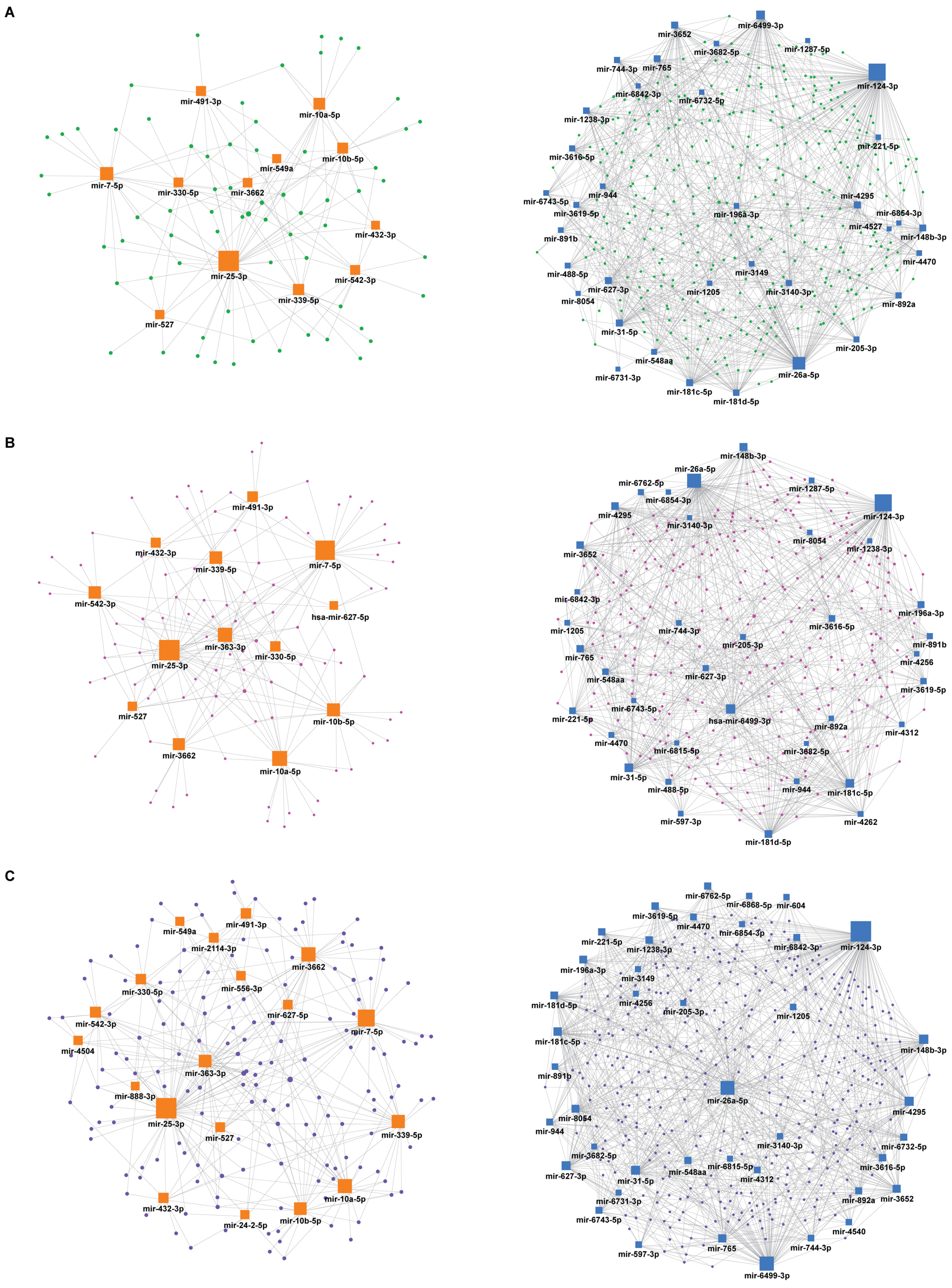
A
mir-491-3p
mir-10a-5p
mir-10b-5p
mir-549a
mir-7-5p
mir-330-5p
mir-3662
mir-432-3p
mir-25-3p
mir-542-3p
mir-339-5p
mir-527
mir-3652
mir-6499-3p
mir-3682-5p
mir-1287-5p
mir-744-3p
mir-765
mir-124-3p
mir-6842-3p
mir-6732-5p
mir-1238-3p
mir-221-5p
mir-3616-5p
mir-944
mir-6743-5p
mir-3619-5p
mir-196a-3p
mir-4295
mir-6854-3p
mir-891b
mir-4527
mir-148b-3p
mir-4470
mir-488-5p
mir-3149
mir-627-3p
mir-1205
mir-3140-3p
mir-8054
mir-892a
mir-31-5p
mir-205-3p
mir-548aa
mir-26a-5p
mir-6731-3p
mir-181c-5p
mir-181d-5p
B
mir-491-3p
mir-432-3p
mir-339-5p
mir-7-5p
mir-542-3p
hsa-mir-627-5p
mir-363-3p
mir-25-3p
mir-330-5p
mir-527
mir-10b-5p
mir-3662
mir-10a-5p
mir-148b-3p
mir-26a-5p
mir-6762-5p
mir-1287-5p
mir-6854-3p
mir-124-3p
mir-4295
mir-3140-3p
mir-8054
mir-1238-3p
mir-3652
mir-6842-3p
mir-196a-3p
mir-1205
mir-744-3p
mir-3616-5p
mir-205-3p
mir-891b
mir-765
mir-4256
mir-548aa
mir-627-3p
mir-3619-5p
mir-6743-5p
hsa-mir-6499-3p
mir-221-5p
mir-892a
mir-4312
mir-4470
mir-6815-5p
mir-3682-5p
mir-31-5p
mir-488-5p
mir-944
mir-181c-5p
mir-597-3p
mir-4262
mir-181d-5p
C
mir-549a
mir-491-3p
mir-2114-3p
mir-3662
mir-330-5p
mir-556-3p
mir-542-3p
mir-627-5p
mir-7-5p
mir-4504
mir-363-3p
mir-888-3p
mir-25-3p
mir-527
mir-339-5p
mir-10a-5p
mir-432-3p
mir-24-2-5p
mir-10b-5p
mir-6762-5p
mir-6868-5p
mir-604
mir-3619-5p
mir-4470
mir-6854-3p
mir-221-5p
mir-1238-3p
mir-6842-3p
mir-124-3p
mir-196a-3p
mir-3149
mir-181d-5p
mir-4256
mir-205-3p
mir-1205
mir-181c-5p
mir-148b-3p
mir-891b
mir-26a-5p
mir-8054
mir-4295
mir-944
mir-3140-3p
mir-3682-5p
mir-6732-5p
mir-6815-5p
mir-3616-5p
mir-627-3p
mir-31-5p
mir-548aa
mir-4312
mir-6731-3p
mir-892a
mir-3652
mir-6743-5p
mir-4540
mir-765
mir-744-3p
mir-597-3p
mir-6499-3p

**Figure S6. miRNet gene networks showing miRNA identified as regulators of CNNM4 against genes from miRTarBase enriched for disease phenotypes from DisGeNET. Related to Figure 5.** Square nodes represent individual miRNA and circles represent genes reported to be linked to disease. Larger node size indicates greater connectivity within the network. Orange squares (leftmost networks) represent downregulating miRNAs and blue squares (rightmost networks) represent upregulating miRNAs. A) miRNET gene networks enriched for liver carcinoma and cirrhosis phenotypes from DisGeNET (green dots). B) miRNET gene networks enriched for benign and malignant mammary neoplasms from DisGeNET (pink dots). C) miRNet gene network enriched for phenotypes common in developmental cognitive disorders from DisGeNET (purple dots).

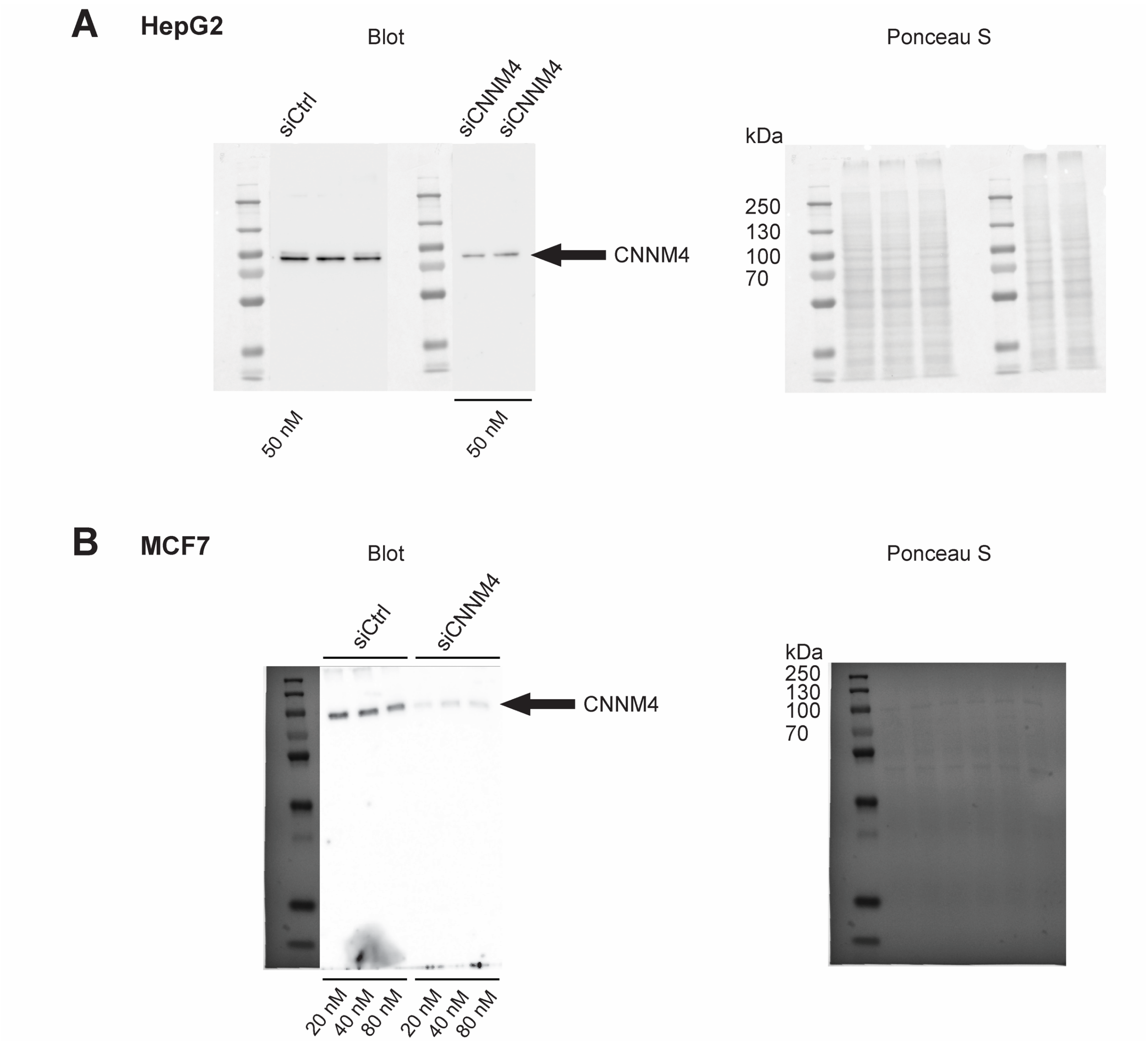


**Figure S7: Validation of antibody for CNNM4.** A) Western blot analysis of HepG2 cells treated with siCNNM4. HepG2 cells were transfected with 50 nM of siCNNM5 for 48 h prior to Western blot analysis. B) Western blot analysis of MCF7 cells titrated with varying concentrations of siCNNM4 (20, 40 and 80 nM) for 48 h prior to Western blot analysis.

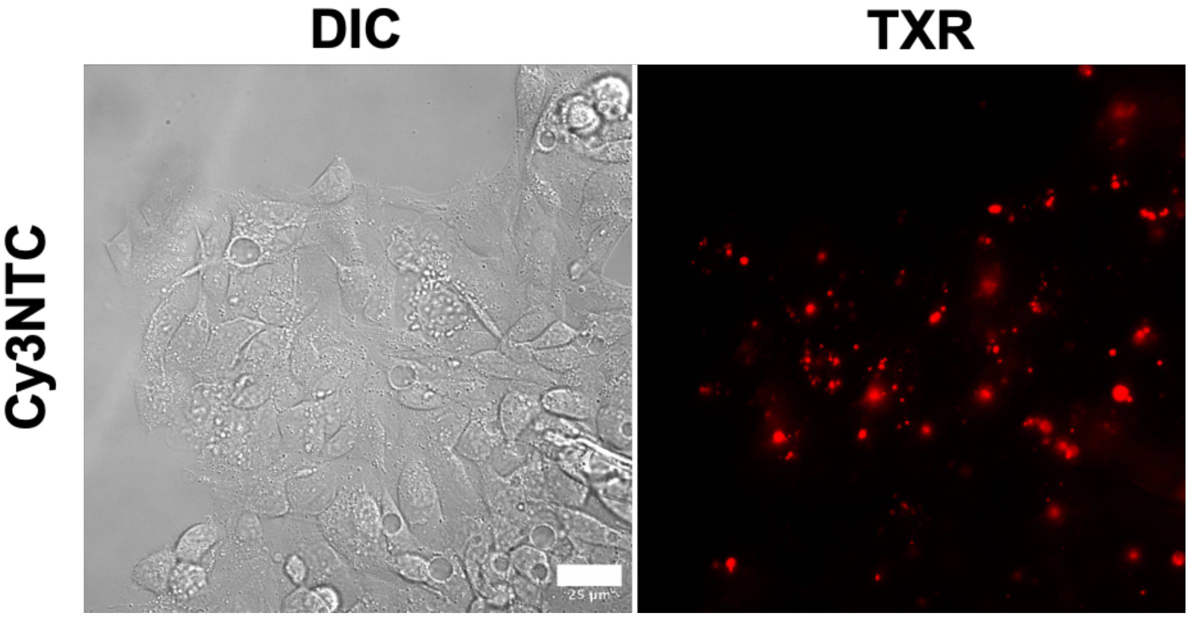


**Figure S8: Confirmation of Dharmacon transfection with Dharmafect 1 in HepG2 cells.** Briefly, HepG2 cells were transfected with a control Cy3-NTC in order to assess if transfection protocol led to uptake of miRNA mimics and anti-miRNAs. Scale bar = 25 μm.
